# Crystal structure of poly[[(2,2′-bi­pyridine)manganese(II)]-di-μ-thio­cyanato]

**DOI:** 10.1107/S1600536814024490

**Published:** 2014-11-19

**Authors:** Stefan Suckert, Susanne Wöhlert, Inke Jess, Christian Näther

**Affiliations:** a Institut für Anorganische Chemie, Christian-Albrechts-Universität Kiel, Max-Eyth-Strasse 2, 24118 Kiel, Germany

**Keywords:** crystal structure, coordination polymer, Mn in octa­hedral coordination, bi­pyridine ligand

## Abstract

In the crystal structure of the polymeric title compound, [Mn(NCS)_2_(C_10_H_8_N_2_)]_*n*_, the Mn^II^ cations are coordinated by one chelating 2,2′-bi­pyridine ligand and four thio­cyanate anions (two *N*- and two *S*-coordinating), forming a distorted [MnN_4_S_2_] octa­hedron. The asymmetric unit consists of one manganese cation located on a twofold rotation axis and half of a 2,2′-bi­pyridine ligand, the other half being generated by the same twofold rotation axis, as well as one thio­cyanate anion in a general position. The Mn^II^ cations are linked by two pairs of μ_1,3_-bridging thio­cyanate ligands into chains along the *c* axis; because the N atoms of the 2,2′-bi­pyridine ligands, as well as the N and the S atoms of the thio­cyanate anions, are each *cis*-coordinating, these chains show a zigzag arrangement.

## Related literature   

For the magnetic properties of the title compound, see: Dockum *et al.* (1983 ▶). For general background to this work, see: Näther *et al.* (2013 ▶).
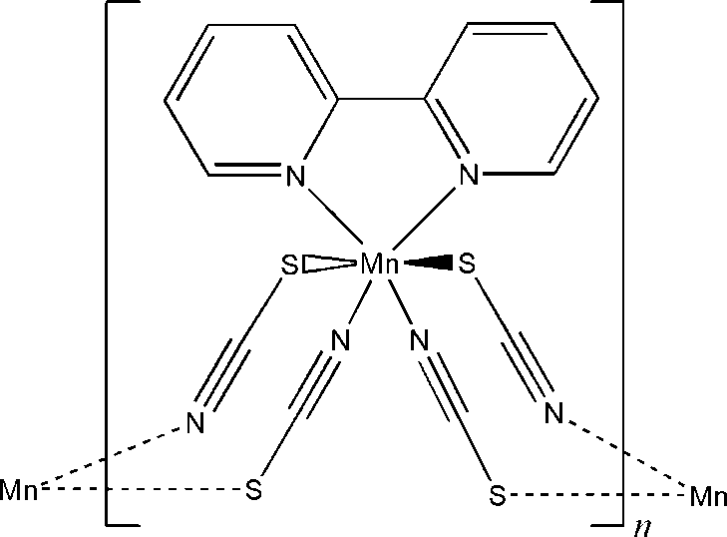


## Experimental   

### Crystal data  

[Mn(NCS)_2_(C_10_H_8_N_2_)]
*M* *_r_* = 327.28
Monoclinic, 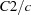

*a* = 7.6158 (5) Å
*b* = 16.2007 (14) Å
*c* = 10.6784 (7) Å
β = 90.129 (8)°
*V* = 1317.51 (17) Å^3^
*Z* = 4
Mo *K*α radiation
μ = 1.31 mm^−1^
*T* = 180 K
0.24 × 0.18 × 0.11 mm

### Data collection  

STOE IPDS-1 diffractometer
Absorption correction: numerical (*X-SHAPE* and *X-RED32*; Stoe & Cie, 2008 ▶) *T*_min_ = 0.714, *T*_max_ = 0.825
5163 measured reflections
1424 independent reflections
1208 reflections with *I* > 2σ(*I*)
*R*_int_ = 0.029

### Refinement  

*R*[*F* ^2^ > 2σ(*F* ^2^)] = 0.026
*wR*(*F* ^2^) = 0.065
*S* = 1.10
1424 reflections
87 parameters
H-atom parameters constrained
Δρ_max_ = 0.22 e Å^−3^
Δρ_min_ = −0.35 e Å^−3^

### 

Data collection: *X-AREA* (Stoe & Cie, 2008 ▶); cell refinement: *X-AREA*; data reduction: *X-AREA*; program(s) used to solve structure: *SHELXS97* (Sheldrick, 2008 ▶); program(s) used to refine structure: *SHELXL97* (Sheldrick, 2008 ▶); molecular graphics: *XP* in *SHELXTL* (Sheldrick, 2008 ▶) and *DIAMOND* (Brandenburg, 1999 ▶); software used to prepare material for publication: *publCIF* (Westrip, 2010 ▶).

## Supplementary Material

Crystal structure: contains datablock(s) I, global. DOI: 10.1107/S1600536814024490/wm5088sup1.cif

Structure factors: contains datablock(s) I. DOI: 10.1107/S1600536814024490/wm5088Isup2.hkl

**Fig. 1.**
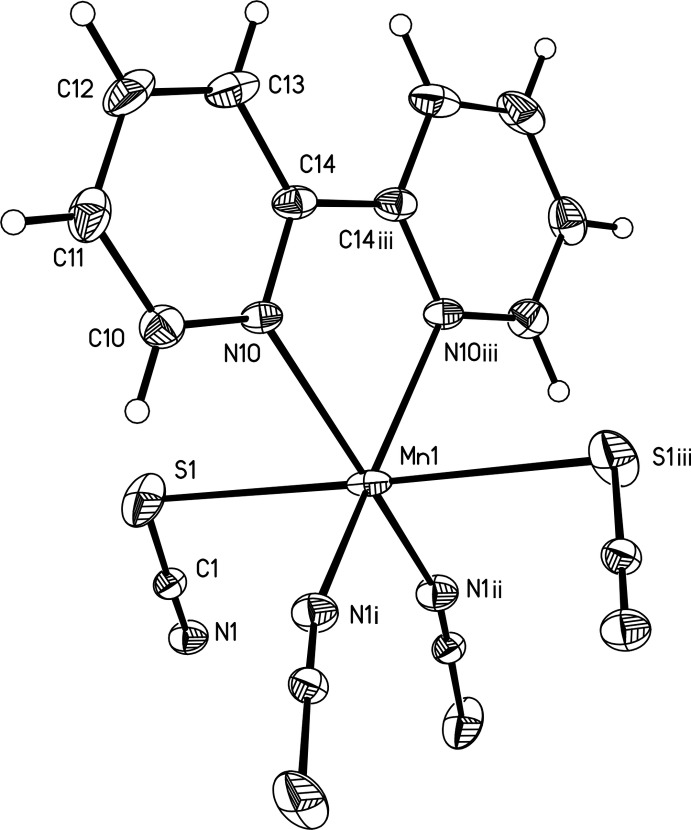
The coordination of the MnII atom in the title compound with atom labelling and displacement ellipsoids drawn at the 50% probability level. [Symmetry codes: i) x,-y+1,z-1/2; ii) -x,-y+1,-z+1; iii) -x,y,-z+1/2.]

**Fig. 2.**
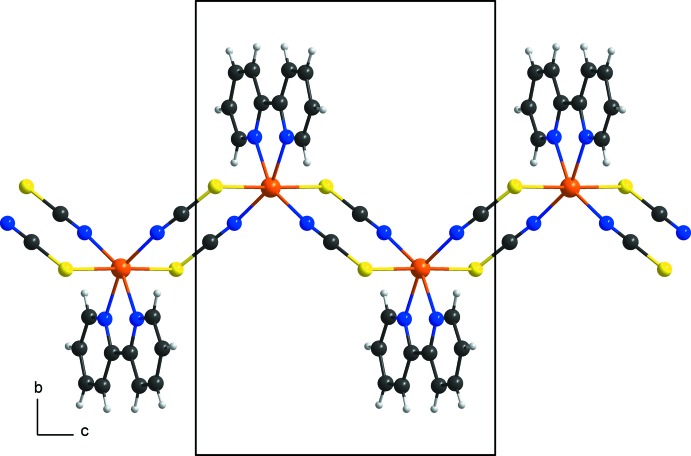
The polymeric arrangement of the chains in the crystal structure of the title compound in a view along the a axis. Colour code: Mn orange; N blue; S yellow; C black; H white.

II . DOI: 10.1107/S1600536814024490/wm5088fig1.tif
The coordination of the Mn^II^ atom in the title compound with atom labelling and displacement ellipsoids drawn at the 50% probability level. [Symmetry codes: i) x,-y+1,z-1/2; ii) −x,-y+1,-z+1; iii) −x,y,-z+1/2.]

a . DOI: 10.1107/S1600536814024490/wm5088fig2.tif
The polymeric arrangement of the chains in the crystal structure of the title compound in a view along the *a* axis. Colour code: Mn orange; N blue; S yellow; C black; H white.

CCDC reference: 1033178

Additional supporting information:  crystallographic information; 3D view; checkCIF report

## Figures and Tables

**Table 1:** Selected bond lengths ()

Mn1N1^i^	2.1318(13)
Mn1N10	2.2433(12)
Mn1S1	2.8138(5)

Symmetry code: (i) 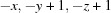.

## supplementary crystallographic information

### S1. Synthesis and crystallization

MnSO_4_·H_2_O was purchased from Merck and 2,2'-bi­pyridine and Ba(NCS)_2_·3 H_2_O were purchased from Alfa Aesar. Mn(NCS)_2_ was synthesized by stirring 17.97 g (58.44 mmol) Ba(NCS)_2_·3H_2_O and 9.88 g (58.44 mmol) MnSO_4_·H_2_O in 300 ml water at RT for three hours. The white precipitate of BaSO_4_ was filtered off and the solvent removed with a rotary evaporator. The homogeneity of the product was investigated by X-ray powder diffraction and elemental analysis. The title compound was prepared by the reaction of (0.4 mmol) 70.0 mg Mn(NCS)_2_ and (0.05 mmol) 7.0 mg 2,2'-bi­pyridine in 1.0 ml aceto­nitrile at RT. After few days, yellow block-shaped crystals of the title compound were obtained.

### S2. Refinement

The H atoms were positioned with idealized geometry and were refined with C—H = 0.93 Å and *U*_eq_(H) = 1.2*U*_eq_(C) using a riding model.

### Crystal data

**Table:**

[Mn(NCS)_2_(C_10_H_8_N_2_)]	*F*(000) = 660
*M**_r_* = 327.28	*D*_x_ = 1.650 Mg m^−^^3^
Monoclinic, *C*2/*c*	Mo *K*α radiation, λ = 0.71073 Å
Hall symbol: -C 2yc	Cell parameters from 5163 reflections
*a* = 7.6158 (5) Å	θ = 3.2–27.0°
*b* = 16.2007 (14) Å	µ = 1.31 mm^−^^1^
*c* = 10.6784 (7) Å	*T* = 180 K
β = 90.129 (8)°	Block, yellow
*V* = 1317.51 (17) Å^3^	0.24 × 0.18 × 0.11 mm
*Z* = 4	

### Data collection

**Table:**

STOE IPDS-1 diffractometer	1424 independent reflections
Radiation source: fine-focus sealed tube	1208 reflections with *I* > 2σ(*I*)
Graphite monochromator	*R*_int_ = 0.029
phi scans	θ_max_ = 27.0°, θ_min_ = 3.2°
Absorption correction: numerical (*X-SHAPE* and *X-RED32*; Stoe & Cie, 2008)	*h* = −9→9
*T*_min_ = 0.714, *T*_max_ = 0.825	*k* = −20→20
5163 measured reflections	*l* = −13→13

### Refinement

**Table:**

Refinement on *F*^2^	Primary atom site location: structure-invariant direct methods
Least-squares matrix: full	Secondary atom site location: difference Fourier map
*R*[*F*^2^ > 2σ(*F*^2^)] = 0.026	Hydrogen site location: inferred from neighbouring sites
*wR*(*F*^2^) = 0.065	H-atom parameters constrained
*S* = 1.10	*w* = 1/[σ^2^(*F*_o_^2^) + (0.041*P*)^2^ + 0.0505*P*] where *P* = (*F*_o_^2^ + 2*F*_c_^2^)/3
1424 reflections	(Δ/σ)_max_ = 0.001
87 parameters	Δρ_max_ = 0.22 e Å^−^^3^
0 restraints	Δρ_min_ = −0.35 e Å^−^^3^

### Special details

**Table:**

Geometry. All esds (except the esd in the dihedral angle between two l.s. planes) are estimated using the full covariance matrix. The cell esds are taken into account individually in the estimation of esds in distances, angles and torsion angles; correlations between esds in cell parameters are only used when they are defined by crystal symmetry. An approximate (isotropic) treatment of cell esds is used for estimating esds involving l.s. planes.
Refinement. Refinement of F^2^ against ALL reflections. The weighted R-factor wR and goodness of fit S are based on F^2^, conventional R-factors R are based on F, with F set to zero for negative F^2^. The threshold expression of F^2^ > 2sigma(F^2^) is used only for calculating R-factors(gt) etc. and is not relevant to the choice of reflections for refinement. R-factors based on F^2^ are statistically about twice as large as those based on F, and R- factors based on ALL data will be even larger.

### Fractional atomic coordinates and isotropic or equivalent isotropic displacement parameters (Å^2^)

**Table:**

	*x*	*y*	*z*	*U*_iso_*/*U*_eq_	
Mn1	0.0000	0.589163 (17)	0.2500	0.01511 (12)	
S1	0.26426 (6)	0.59114 (3)	0.43456 (4)	0.02879 (14)	
C1	0.1959 (2)	0.53098 (9)	0.54809 (13)	0.0157 (3)	
N1	0.14566 (19)	0.48959 (8)	0.62863 (13)	0.0191 (3)	
N10	0.15848 (17)	0.70040 (7)	0.19663 (11)	0.0150 (3)	
C10	0.3171 (2)	0.69584 (10)	0.14411 (14)	0.0205 (3)	
H10	0.3650	0.6440	0.1287	0.025*	
C11	0.4133 (2)	0.76537 (11)	0.11149 (15)	0.0246 (4)	
H11	0.5240	0.7605	0.0756	0.029*	
C12	0.3400 (2)	0.84198 (11)	0.13384 (16)	0.0277 (4)	
H12	0.4008	0.8897	0.1122	0.033*	
C13	0.1770 (3)	0.84748 (9)	0.18819 (16)	0.0256 (4)	
H13	0.1267	0.8988	0.2037	0.031*	
C14	0.0878 (2)	0.77516 (9)	0.21990 (13)	0.0175 (3)	

### Atomic displacement parameters (Å^2^)

**Table:**

	*U*^11^	*U*^22^	*U*^33^	*U*^12^	*U*^13^	*U*^23^
Mn1	0.0203 (2)	0.00785 (16)	0.01723 (18)	0.000	0.00916 (12)	0.000
S1	0.0261 (3)	0.0358 (3)	0.0244 (2)	−0.01473 (18)	0.00026 (18)	0.01435 (16)
C1	0.0150 (8)	0.0134 (6)	0.0186 (7)	−0.0014 (6)	0.0007 (5)	−0.0002 (5)
N1	0.0218 (7)	0.0167 (6)	0.0187 (6)	−0.0016 (5)	0.0031 (5)	0.0031 (5)
N10	0.0193 (7)	0.0116 (5)	0.0141 (6)	−0.0013 (5)	0.0024 (5)	0.0023 (4)
C10	0.0215 (9)	0.0203 (7)	0.0195 (7)	−0.0010 (6)	0.0016 (6)	0.0026 (6)
C11	0.0196 (9)	0.0319 (8)	0.0222 (8)	−0.0067 (7)	−0.0002 (6)	0.0060 (7)
C12	0.0319 (11)	0.0242 (8)	0.0269 (9)	−0.0145 (7)	−0.0025 (7)	0.0075 (6)
C13	0.0352 (11)	0.0140 (7)	0.0275 (8)	−0.0055 (7)	−0.0005 (7)	0.0002 (6)
C14	0.0254 (9)	0.0133 (7)	0.0137 (6)	−0.0011 (6)	−0.0009 (6)	0.0018 (5)

### Geometric parameters (Å, º)

**Table:**

Mn1—N1^i^	2.1318 (13)	N10—C14	1.3485 (18)
Mn1—N1^ii^	2.1318 (13)	C10—C11	1.389 (2)
Mn1—N10^iii^	2.2433 (12)	C10—H10	0.9300
Mn1—N10	2.2433 (12)	C11—C12	1.382 (3)
Mn1—S1^iii^	2.8138 (5)	C11—H11	0.9300
Mn1—S1	2.8138 (5)	C12—C13	1.375 (3)
S1—C1	1.6411 (15)	C12—H12	0.9300
C1—N1	1.156 (2)	C13—C14	1.396 (2)
N1—Mn1^ii^	2.1318 (13)	C13—H13	0.9300
N10—C10	1.335 (2)	C14—C14^iii^	1.486 (3)
			
N1^i^—Mn1—N1^ii^	106.48 (7)	C10—N10—C14	119.27 (13)
N1^i^—Mn1—N10^iii^	156.38 (5)	C10—N10—Mn1	123.37 (11)
N1^ii^—Mn1—N10^iii^	92.60 (5)	C14—N10—Mn1	117.36 (10)
N1^i^—Mn1—N10	92.60 (5)	N10—C10—C11	122.62 (16)
N1^ii^—Mn1—N10	156.38 (5)	N10—C10—H10	118.7
N10^iii^—Mn1—N10	73.10 (7)	C11—C10—H10	118.7
N1^i^—Mn1—S1^iii^	87.33 (4)	C12—C11—C10	118.14 (16)
N1^ii^—Mn1—S1^iii^	93.46 (4)	C12—C11—H11	120.9
N10^iii^—Mn1—S1^iii^	77.55 (3)	C10—C11—H11	120.9
N10—Mn1—S1^iii^	101.38 (3)	C13—C12—C11	119.78 (15)
N1^i^—Mn1—S1	93.46 (4)	C13—C12—H12	120.1
N1^ii^—Mn1—S1	87.33 (4)	C11—C12—H12	120.1
N10^iii^—Mn1—S1	101.38 (3)	C12—C13—C14	119.23 (15)
N10—Mn1—S1	77.55 (3)	C12—C13—H13	120.4
S1^iii^—Mn1—S1	178.69 (2)	C14—C13—H13	120.4
C1—S1—Mn1	106.45 (6)	N10—C14—C13	120.96 (15)
N1—C1—S1	178.83 (15)	N10—C14—C14^iii^	116.09 (8)
C1—N1—Mn1^ii^	166.59 (14)	C13—C14—C14^iii^	122.95 (10)

Symmetry codes: (i) *x*, −*y*+1, *z*−1/2; (ii) −*x*, −*y*+1, −*z*+1; (iii) −*x*, *y*, −*z*+1/2.
