# Supplementary figures and images for: Crystal structure of poly[[(2,2′-bi­pyridine)manganese(II)]-di-μ-thio­cyanato]
Source: Acta Crystallogr Sect E Struct Rep Online. 2014 Nov 19;70(Pt 12):m401–2. doi: 10.1107/S1600536814024490 (PMC4257407; doi:10.1107/S1600536814024490)

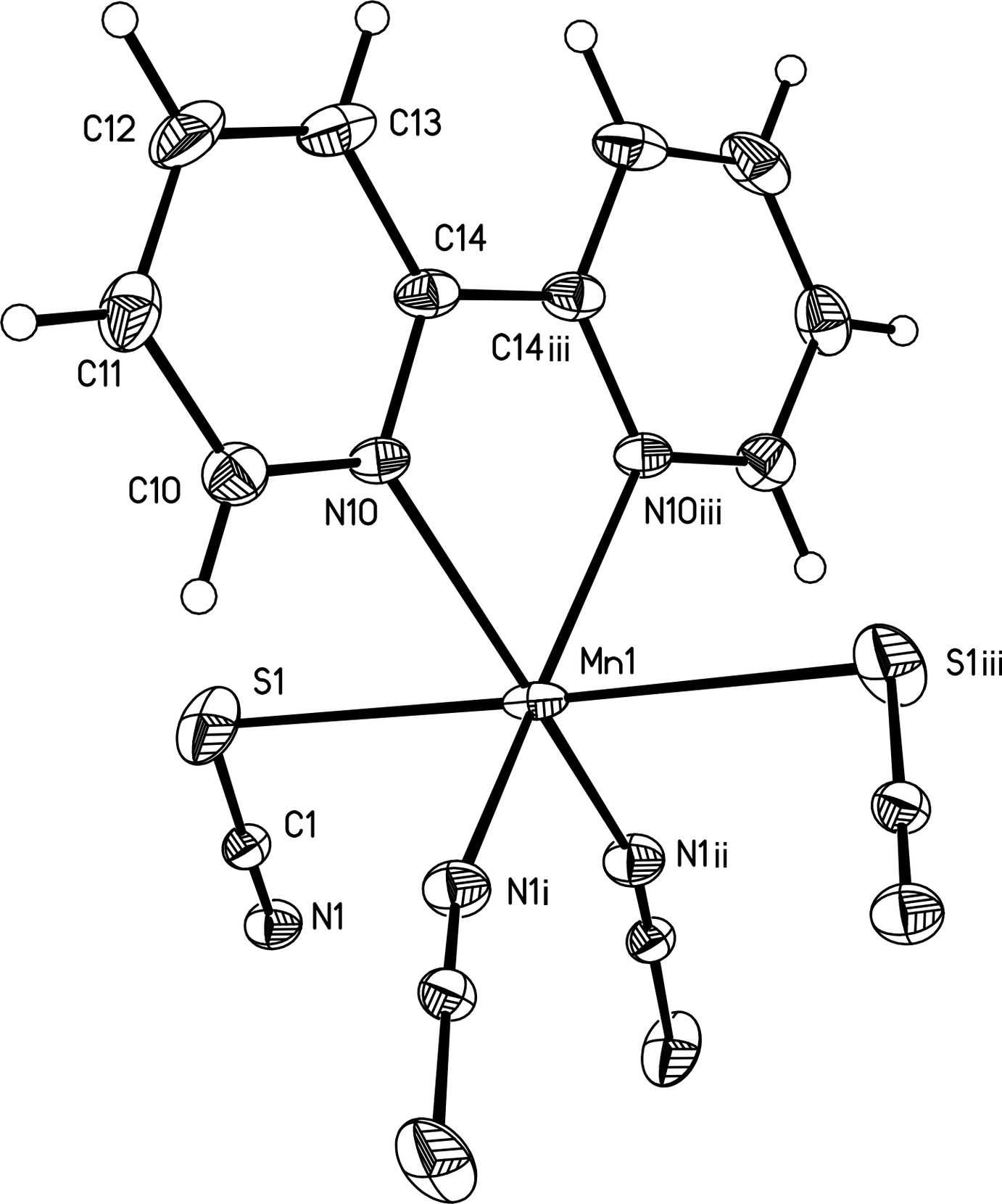

Supplement: Supplementary file 3 [file e-70-0m401-fig1.tif]

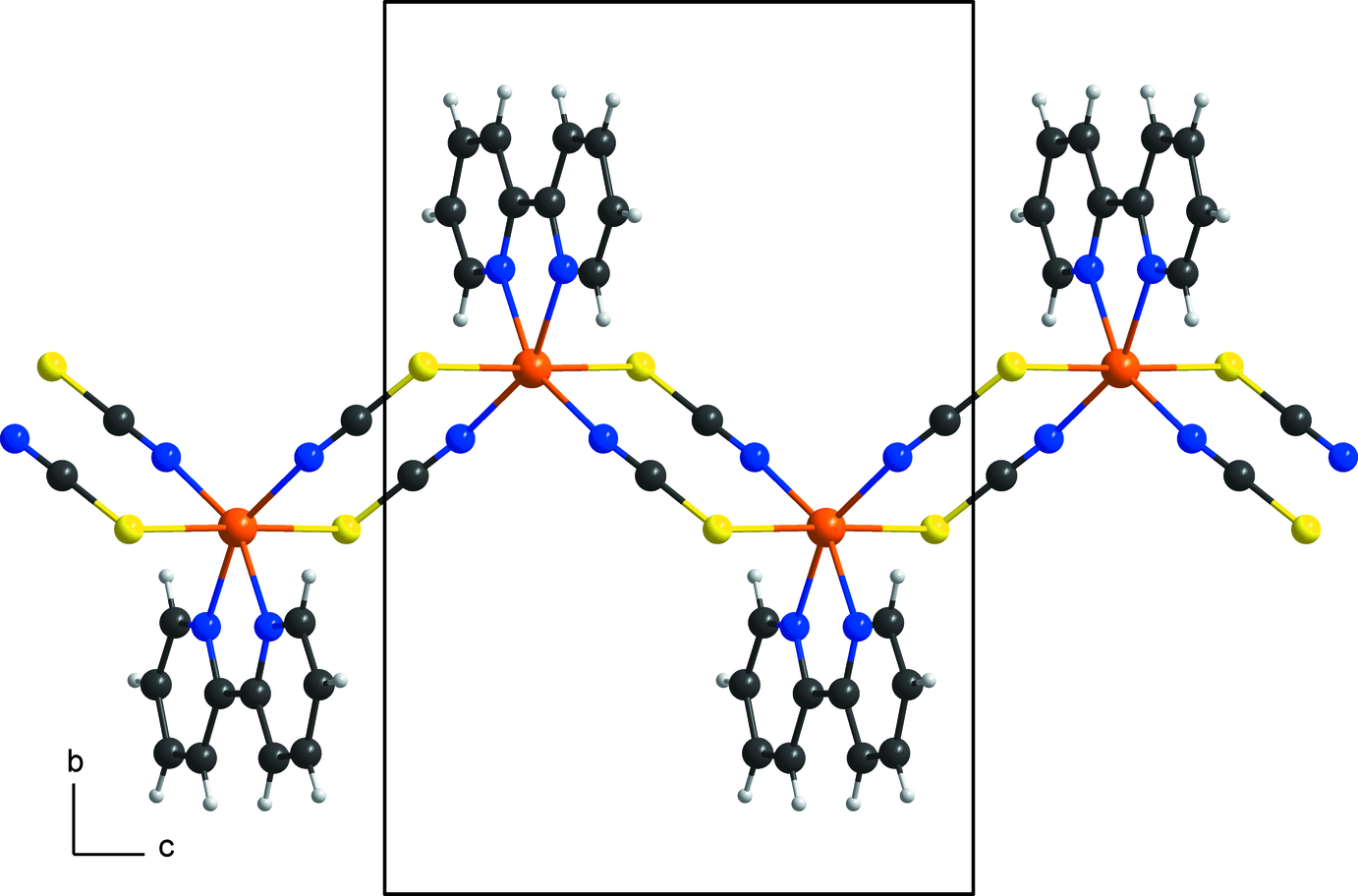

Supplement: Supplementary file 4 [file e-70-0m401-fig2.tif]
